# Supplementary figures and images for: Human bronchial epithelial cell‐derived extracellular vesicle therapy for pulmonary fibrosis via inhibition of TGF‐β‐WNT crosstalk
Source: J Extracell Vesicles. 2021 Aug 2;10(10):e12124. doi: 10.1002/jev2.12124 (PMC8329991; doi:10.1002/jev2.12124)

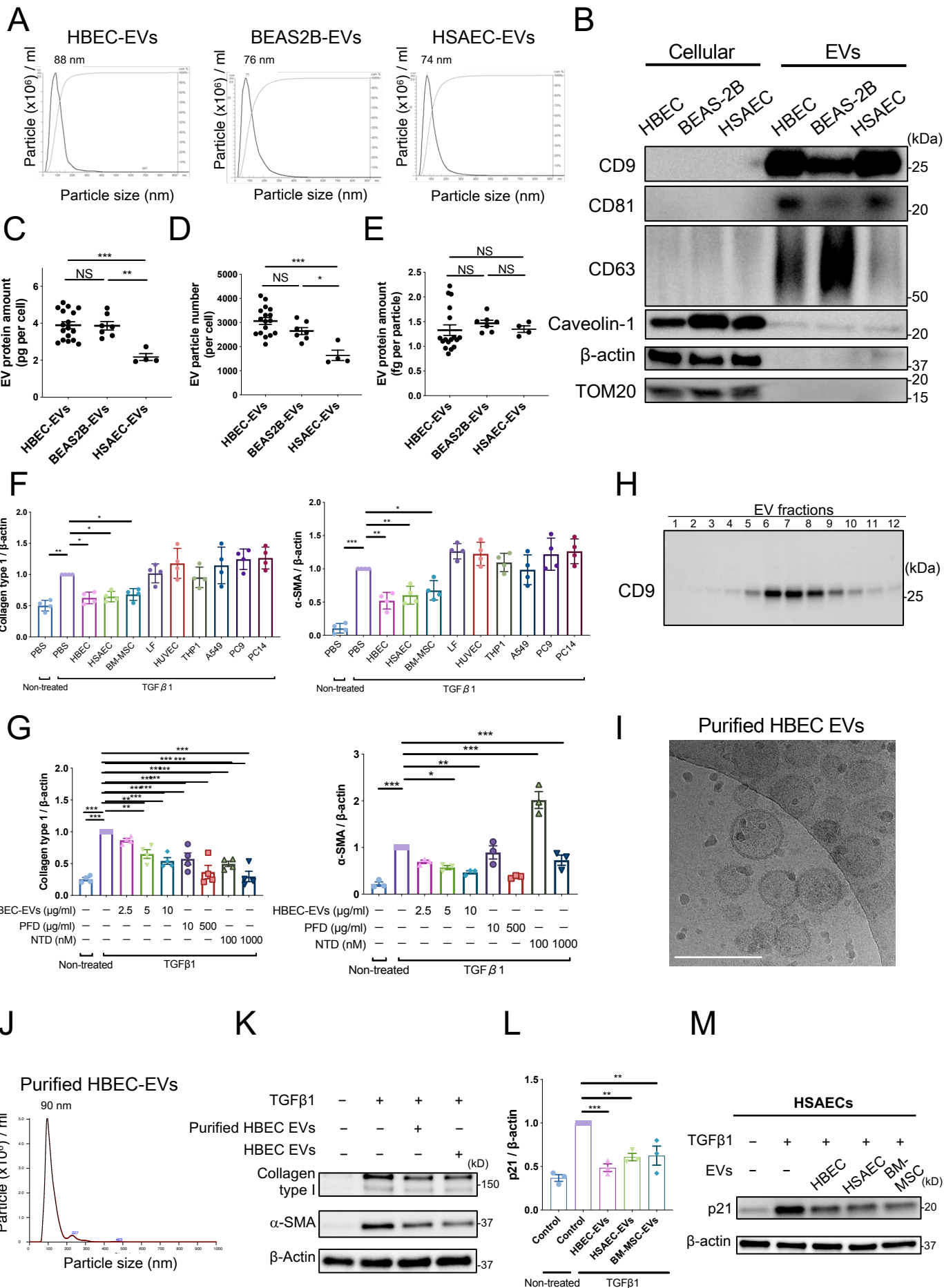

**Fig.S1**

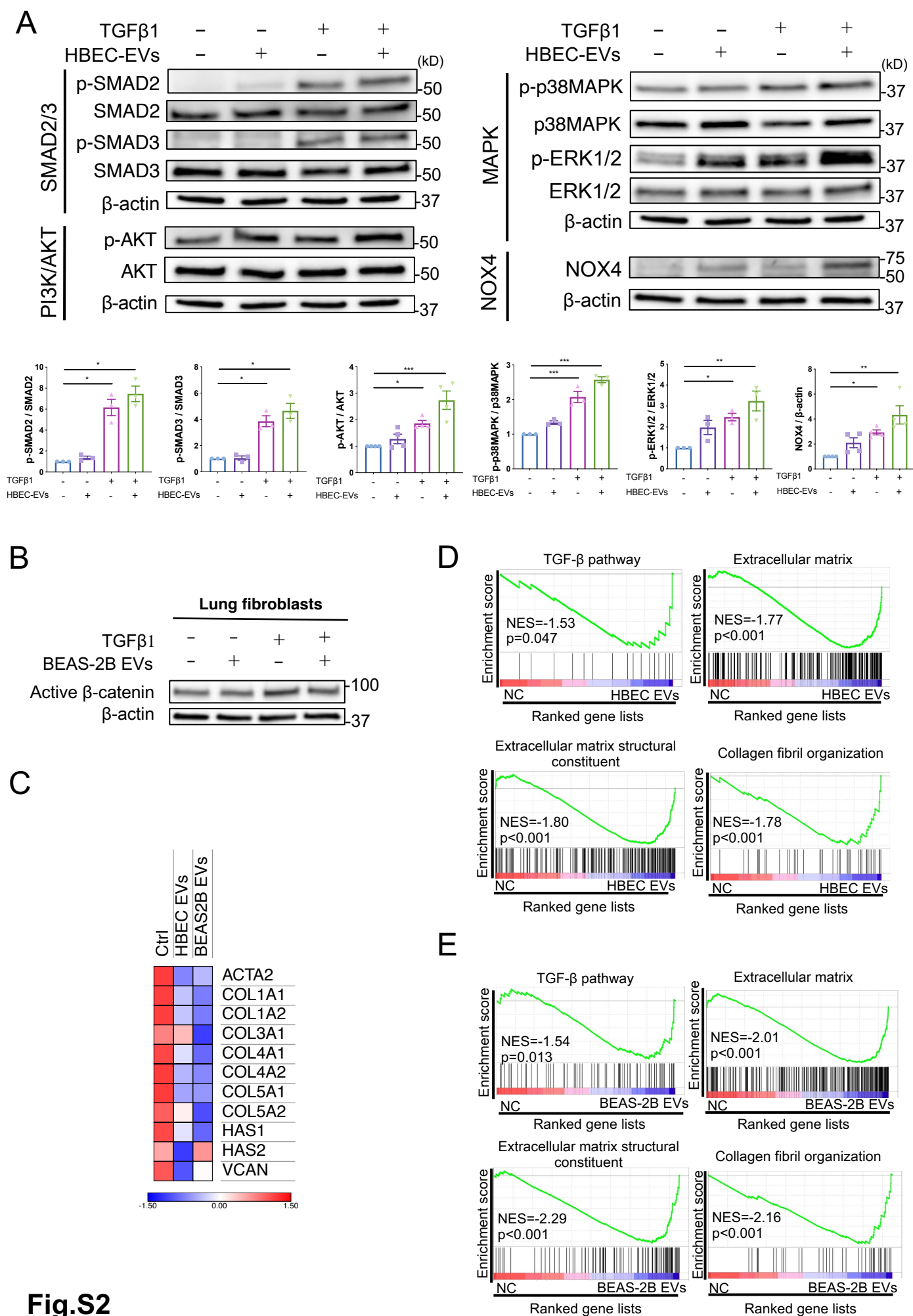

**Fig.S2**

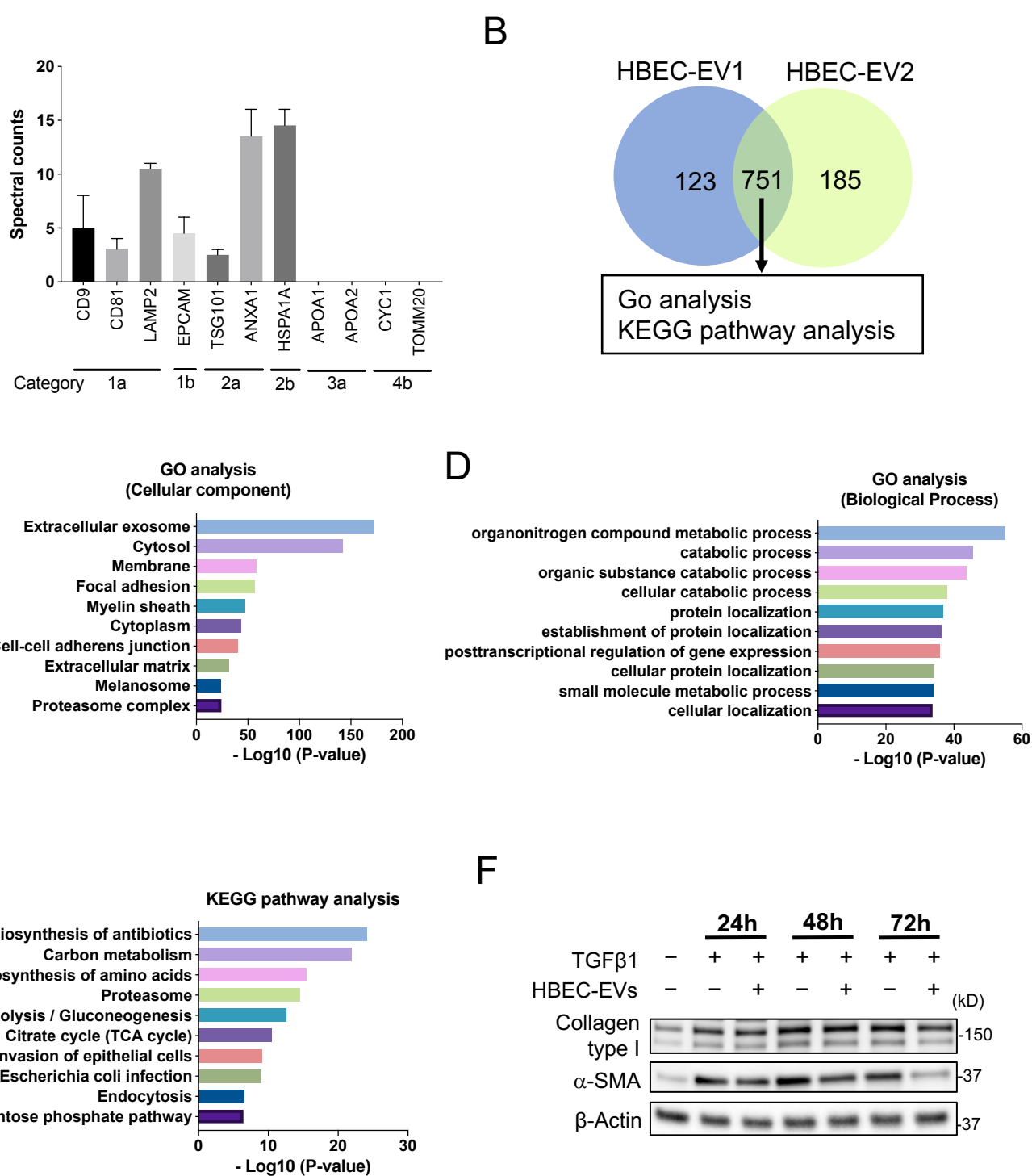

**Fig.S3**

A

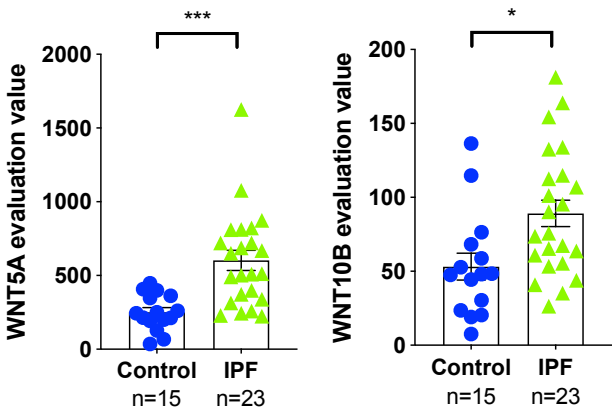

B

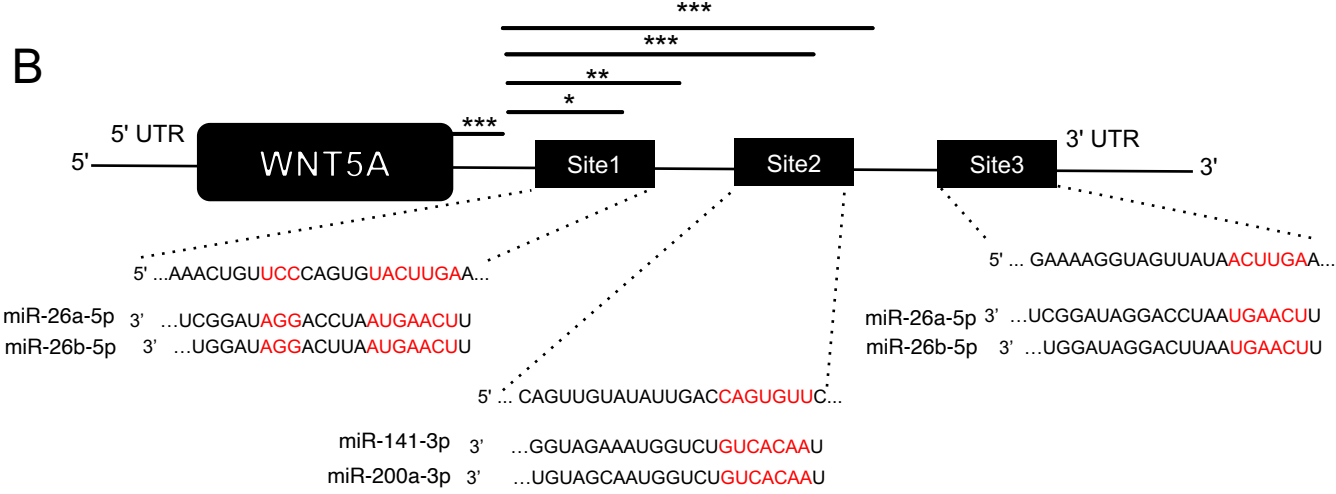

C

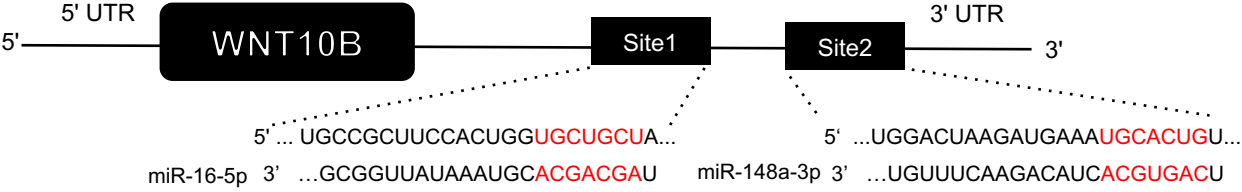

Fig. S4

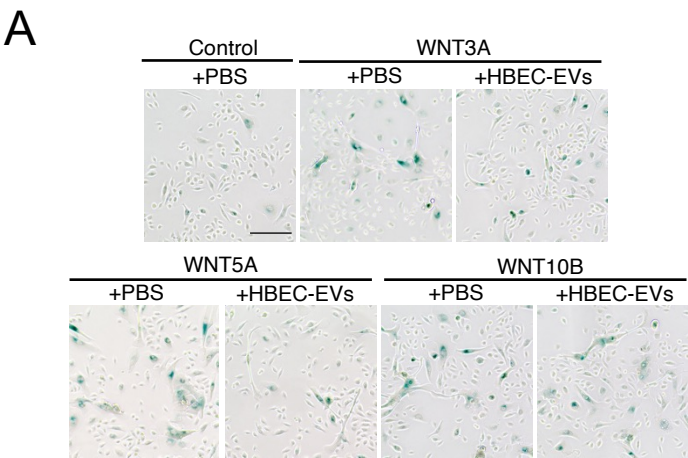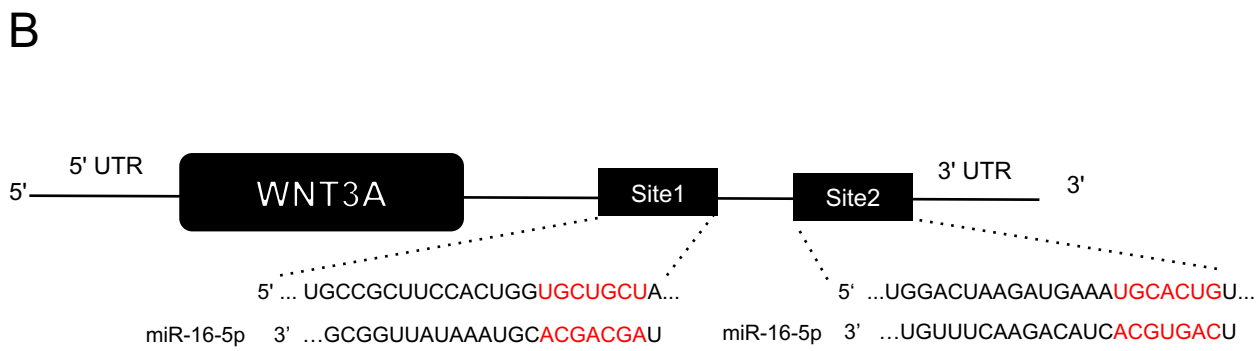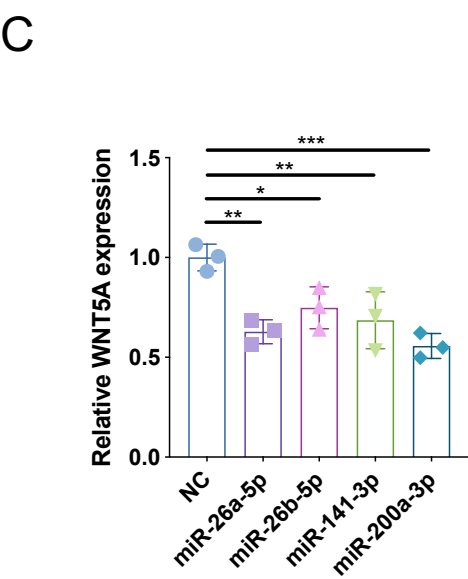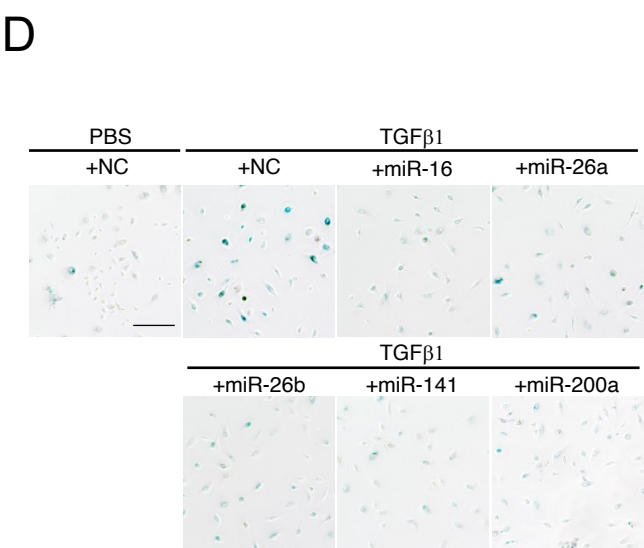

**Fig.S5**

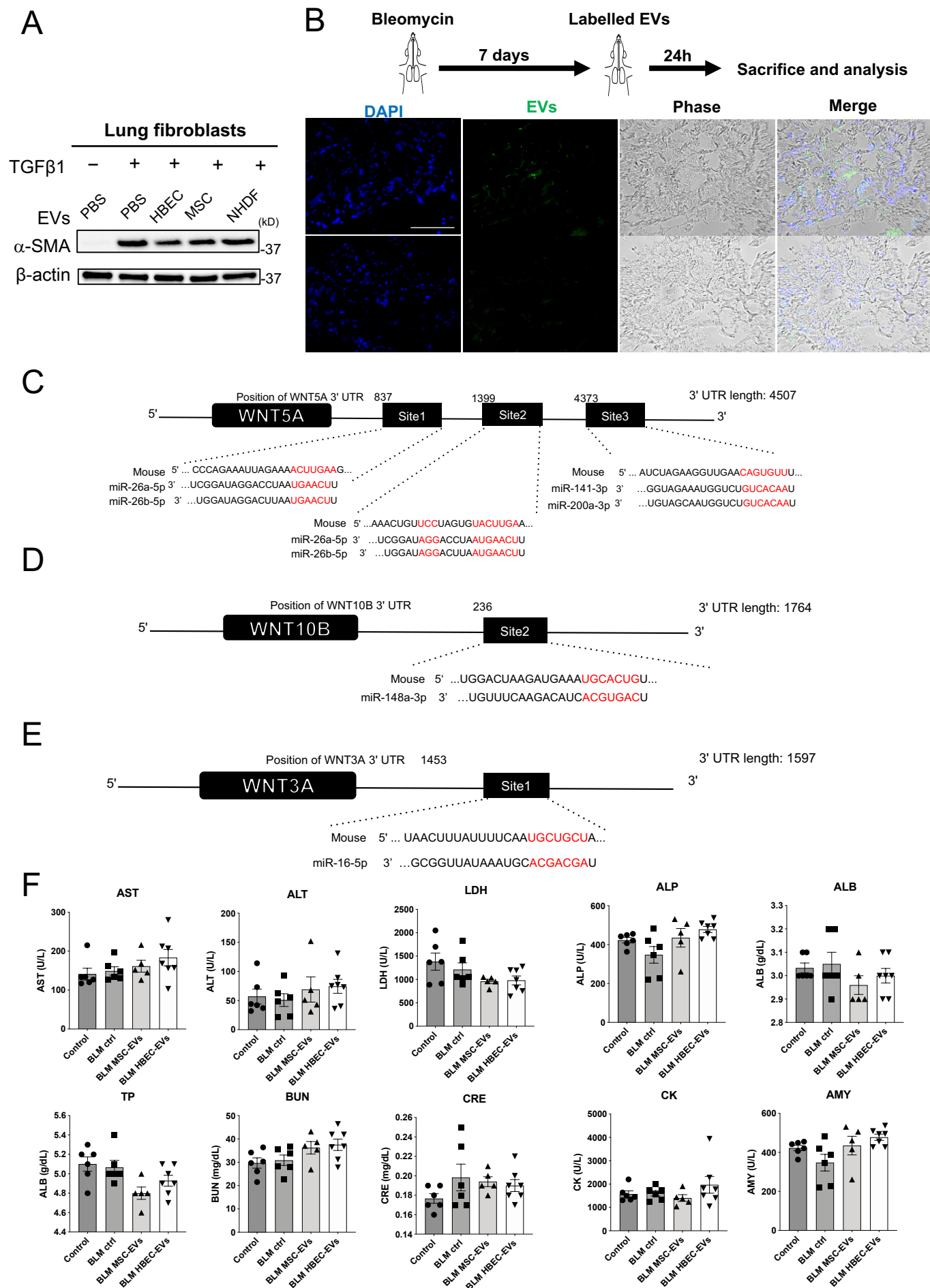

**Fig.S6**

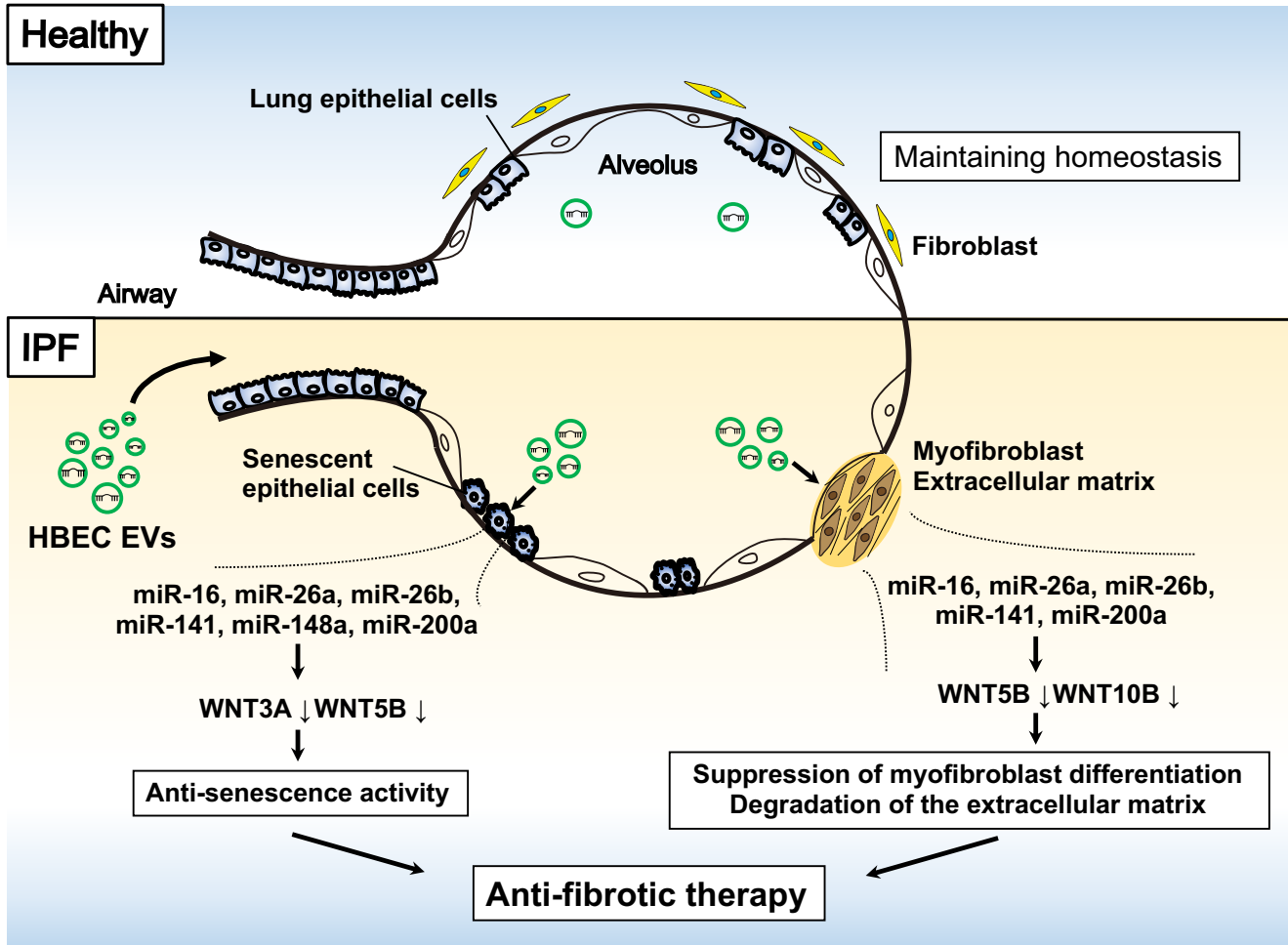

Fig.S7

Supplement: Supplementary file 2 — Supplementary information [file JEV2-10-e12124-s001.pdf]
